# Behavior of over-reinforced concrete beams including longitudinal voids in the compression zone reinforced with aluminum tubes

**DOI:** 10.1038/s41598-026-52780-5

**Published:** 2026-06-26

**Authors:** Saad A. Yehia, Mohamed Ghalla, Yahia Iskander, Rabeea W. Bazuhair, Yahya M. Bin Mahfouz, Sabry Fayed

**Affiliations:** 1 Civil Engineering Department, Higher Institute of Engineering and Technology, Kafrelsheikh, Egypt; 2 Department of Civil Engineering, Faculty of Engineering, Kafrelsheikh University, Kafr El Sheikh, Egypt; 3 Department of Civil Engineering, College of Engineering and Architecture, Umm Al-Qura University, Makkah, Saudi Arabia

**Keywords:** Over-reinforced concrete beams, Voids, Compression zone, Aluminum tubes, Failures, Ultimate load, Engineering, Materials science

## Abstract

This study investigates the structural performance of over-reinforced concrete (RC) beams incorporating longitudinal compression-zone voids, a solution proposed to improve spatial efficiency for utility integration. However, such voids may significantly reduce strength and promote brittle failure. To address this issue, aluminum tubes (AT) are introduced as internal reinforcement within the voids. An experimental program comprising eleven beams (one solid, five hollow without reinforcement, and five hollow with AT) was conducted. Key parameters included void ratio (3.1–8%), number (single/double), orientation (vertical/horizontal), and AT ratio (0.75–1.8%). Results show that unreinforced voids reduce ultimate load capacity (${P}_{u}$) by up to 36.5% and energy absorption (EA) by 44.8%. In contrast, AT reinforcement significantly enhances performance, increasing ${P}_{u}$ by 57–135% and EA by 126–264%, while improving ductility and delaying brittle crushing. Horizontally oriented ATs achieved superior performance, with up to 10.8% higher ${P}_{u}$ compared to vertical configurations. Increasing AT ratio (≥ 0.75%) further enhanced post-cracking behavior with minimal effect on initial stiffness. The findings demonstrate that aluminum tubes effectively restore and enhance the structural efficiency of voided over-reinforced beams. For practical applications, void ratios up to 8% are recommended with internal AT reinforcement as a lightweight, corrosion-resistant alternative to conventional external strengthening methods.

## Introduction

Utility infrastructure, including water supply, drainage, electricity, and telecommunication lines, is commonly installed beneath reinforced concrete (RC) beams and concealed within dropped ceilings, resulting in spatial inefficiencies due to dead height^1,2^. Embedding longitudinal voids within the RC beam cross-section presents a viable alternative, enabling the direct integration of service lines. This modification can eliminate the dead height and enhance vertical clearance, offering substantial architectural and functional benefits in both residential and office structures.

Behavior of hollow reinforced concrete beams subjected to flexural, shear, and torsional loading has been the focus of several experimental and computational studies. In an experimental investigation, Balaji and Vetturayasudharsanan^3^ assessed how varying the number of circular openings along the longitudinal axis, while maintaining an equal total opening area, influences the flexural behavior of RC beams. The beams, with cross-sectional dimensions of 150 × 200 mm, incorporated circular voids. Findings demonstrated that beams with a single larger opening exhibited superior flexural capacity compared to their counterparts with two smaller openings. Additionally, a recent experimental investigation by Elamary et al.^4^ focused on effect of longitudinal voids on the flexural ability of hollow RC beams. The results showed only minor reductions in failure load when the voids accounted for less than 10% of the total cross-sectional area. Furthermore, a numerical analysis was undertaken to assess the effect of transverse outlet positions—either near the supports or at mid-span—on the beams’ structural performance.

In another study, Manikandan et al.^5^ explored the effect of longitudinal voids in RC beams and reported that a 25% reduction of concrete in the tensile zone had a negligible effect on flexural capacity, deflection, and strain distribution. Hollow beams constructed with high-strength concrete (${f}_{c}$ ranging from 63 to 73 MPa) and reduced cross sections of up to 44.4% were also found to perform comparably to their solid counterparts in terms of toughness and load resistance^6^. Both solid and hollow beams experienced similar crack patterns and failure mechanisms^7–10^. Moreover, hollow ECC beams with opening diameters of 60 mm and 80 mm achieved higher ductility than solid ECC beams, indicating their potential for energy-dissipating applications^11^. However, there is limited experimental data on the performance of hollow RC beams under combined loadings such as bending–torsion^12^ or shear–torsion^13^. Alnuaimi et al.^14^ tested 14 beams to investigate the effect of the torsion-to-bending moment ratio (0.19–2.62), showing that this parameter significantly influences failure loads in both hollow and solid sections. Additionally, the effect of concrete compressive strength (${f}_{c}$ = 46.2–96.7 MPa) and torsional reinforcement ratio (0.30–2.68%) on cracking behavior was examined in^15^.

Most importantly, although significant work has been conducted on void geometry and distribution, very limited attention has been given to internal reinforcement strategies within voids, particularly the use of aluminum sections as an alternative strengthening approach. Apart from the study by Mansour et al.^16^*,* which investigated behavior of concrete beams including interior voids that were reinforced with aluminum sections, no other research has comprehensively addressed this topic. Their findings, based on experimental testing and 3D finite element modeling, demonstrated that aluminum reinforcement improved crack distribution and effectively restored the load-carrying capacity in beams with 16–36% voids. Furthermore, beams with circular voids exhibited superior performance compared to those with square voids, regardless of the reinforcement configuration. The majority of previous efforts to strengthen hollow RC beams mainly rely on external systems such as FRP composites^17,18^ or steel plates^19–21^, achieving notable improvements in ductility and ultimate strength. For instance, Jeyakumar et al.^18^ experimentally investigated eight pairs of solid and hollow RC beams strengthened with steel fibers (SF) and FRP warping. Results showed SF significantly improved load capacity, while FRP alone was less effective. The combined SF–FRP system enhanced strength, enabling hollow beams to achieve comparable performance with reduced material usage. Hii and Al-Mahaidi^22^ investigated torsional strengthening of solid and box-section reinforced concrete beams using externally bonded CFRP through experimental testing and nonlinear finite element analysis. Results demonstrated significant improvements, with increases up to 40% in cracking torque and 78% in ultimate strength. In another study, Bernardo and Lopes^19^ examined torsional behavior of hollow RC beams with unbalanced longitudinal and transverse reinforcement. Results showed insufficient transverse reinforcement governs performance, while excess longitudinal reinforcement has limited effect. External steel stirrups effectively enhanced torsional strength, stiffness, and cracking behavior, although they did not fully match balanced internal reinforcement. Further experimental research is recommended. Al-Smadi et al.^23^ investigated RC beams with longitudinal openings strengthened using CFRP sheets through experiments and nonlinear FE analysis. Openings reduced capacity by up to 22%. CFRP effectively restored strength, achieving gains up to 16%. Ring strips and steel anchors enhanced performance, delaying debonding and improving bond behavior significantly. Nonetheless, the practical implementation of FRP remains limited by its high expense and poor fire performance^24–28^. Moreover, the application of steel plates often entails recurring maintenance due to vulnerability to rust and corrosion, which elevates the total expenditure over time.

Therefore, the present study proposes the use of embedded aluminum tubes as an internal reinforcement system within compression-zone voids of over-reinforced RC beams. Unlike external FRP- or steel-based strengthening methods, aluminum tubes act as an integral structural component within the beam, offering a combined advantage of a high strength-to-weight ratio, corrosion resistance, and improved stress redistribution in the compression zone. Furthermore, most previous studies have focused on under-reinforced or normally reinforced beams, while over-reinforced beams with compression-zone voids remain largely unexplored, despite their susceptibility to brittle failure due to premature concrete crushing prior to steel yielding. Accordingly, this study investigates the flexural behavior of over-reinforced RC beams containing longitudinal voids reinforced internally with aluminum tubes, aiming to quantify their effectiveness in restoring strength, enhancing ductility, and mitigating brittle failure mechanisms.

## 2. Research significance

Creating openings in the concrete beams has become a necessity in the concrete buildings. These openings are used to pass electromechanical service pipes, sewage pipes, etc. Many researchers have studied the effect of transverse openings in the beams, while research on longitudinal openings is scarce. Therefore, this study focused on longitudinal openings in the concrete beams, especially in the compression zone. The main aim of this study is to experimentally study structural performance of over-reinforced concrete (RC) beams containing voids in the compression zone, both with and without internal reinforcement using embedded aluminum sections. A total of eleven RC beams were examined, including one solid beam, five hollow beams without internal reinforcement, and five hollow beams internally reinforced with aluminum tubes. The study systematically explores the influence of void ratio, number, orientation, and the presence of internal aluminum reinforcement on overall beam performance. The research aims to evaluate the feasibility of using embedded aluminum sections as a sustainable and cost-effective alternative to conventional external strengthening methods, such as fiber-reinforced polymers (FRP) or steel plates. This work contributes valuable insights for future design codes and encourages further exploration of aluminum-reinforced structural elements in modern construction.

## Experimental work

### Items used

#### Concrete

The control concrete mixture (NC) was proportioned to achieve normal strength and adequate workability, as summarized in Table 1. The mix consisted of 300 kg/m^3^ of cement and 150 kg/m^3^ of water, corresponding to a water–cement ratio of 0.50. Fine and coarse aggregates were incorporated at 650 kg/m^3^ and 1290 kg/m^3^, respectively, ensuring proper grading and mechanical stability of the mix. To enhance workability without increasing the water content, a superplasticizer dosage of 12 kg/m^3^ was used. This balanced mix design provided a reliable baseline for evaluating the structural performance of the tested specimens, ensuring consistency in material properties and minimizing variability in experimental results. To characterize the mechanical properties, standard specimens were cast alongside the test beams and cured under identical conditions. The compressive strength was determined from the average of three 150 × 150 × 150 mm cube specimens, while the tensile strength was obtained from splitting tensile tests on standard cylinders. The average compressive strength of concrete was 30 MPa, and the corresponding splitting tensile strength was approximately 2 MPa. Testing was conducted in accordance with the Egyptian Code of Practice (ECP)^29^.

**Table 1:** Elements of mixture used.

Concrete mix type	Cement ($kg/{m}^{3}$)	Water $(kg/{m}^{3})$	Fine aggregate ($kg/{m}^{3}$)	Coarse aggregate ($kg/{m}^{3}$)	Super plasticizer ($kg/{m}^{3}$)	Water/cement (%)
NC	300	150	650	1290	12	0.50

#### Reinforcement bars

**Fig. 1:**
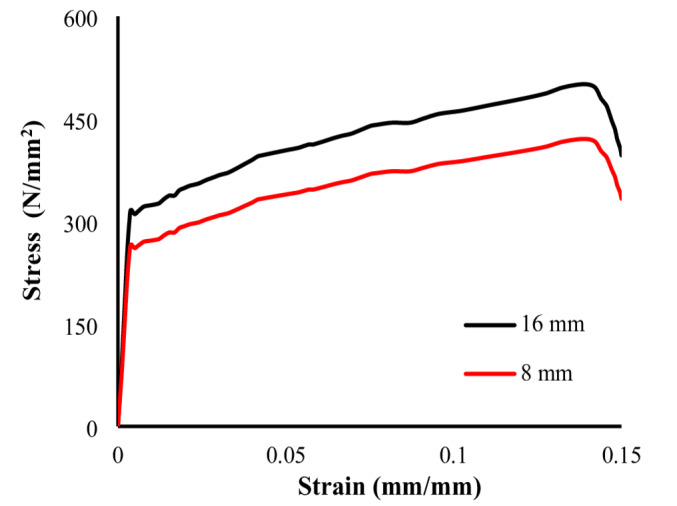
Stress–strain curves of the reinforcing bars.

In this study, two rebars were used for reinforcing the beams (16 mm and 8 mm). Tension experiments were conducted on rebars used and the findings were listed in Table 2. Also, Fig. 1 shows tensile stress–strain response of these bars. 12 mm diameter rebars showed ultimate/yield stresses of 680 MPa and 500 MPa. 8 mm diameter rebars showed ultimate/yield stresses of 340 MPa and 250 MPa.

**Table 2:** Bars used.

Diameter (mm)	Face features	Poisson’s ratio	Elasticity factor (GPa)	Yield (MPa)	Maximum (MPa)	Elongation (%)
8	Smoothed	0.3	201	255.6	343.9	39
16	Ribbed	0.3	201	520.6	679.5	25

#### Aluminum tube

**Fig. 2:**
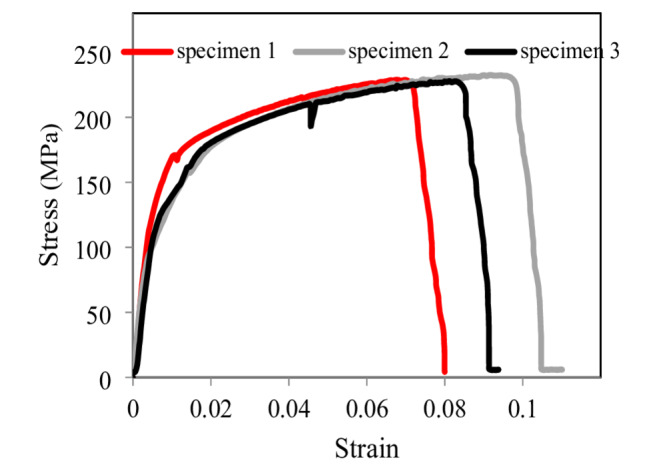
Tension curves of aluminum plates.

The aluminum alloy tubes (AT) used in this study were supplied by Misr Aluminum Company, Egypt. A thermally insulated aluminum alloy was selected for this investigation. To evaluate the mechanical properties of the AT, uniaxial tensile tests were conducted on three specimens. The test specimens were cut from the aluminum tubes and prepared in accordance with ASTM B928/B928M-09^30^. Tension experiments were performed using a 200 kN testing device. Figure 2 shows stress–strain curves of AT. Key mechanical properties are summarized in Table 3. The mean yield stress of AT was 115 MPa and its ultimate strength was 230 MPa.

**Table 3:** Findings ATs.

Samples	Elongation %	Yield strength (MPa)	Ultimate strength (MPa)
Sample 1	9	122	228
Sample 2	10.58	103	233
Sample 3	9.87	110	228
Mean	9.14	115	230

### RC beam specimen characteristics

**Fig. 3:**
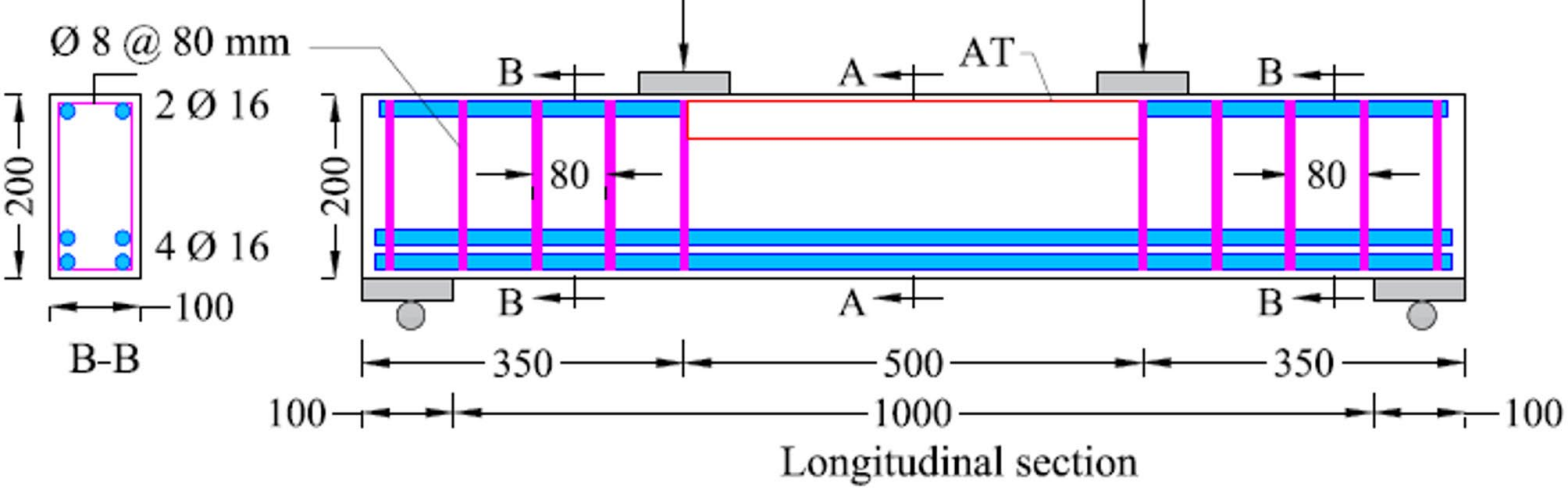
General details of all beams (mm).

**Fig. 4:**
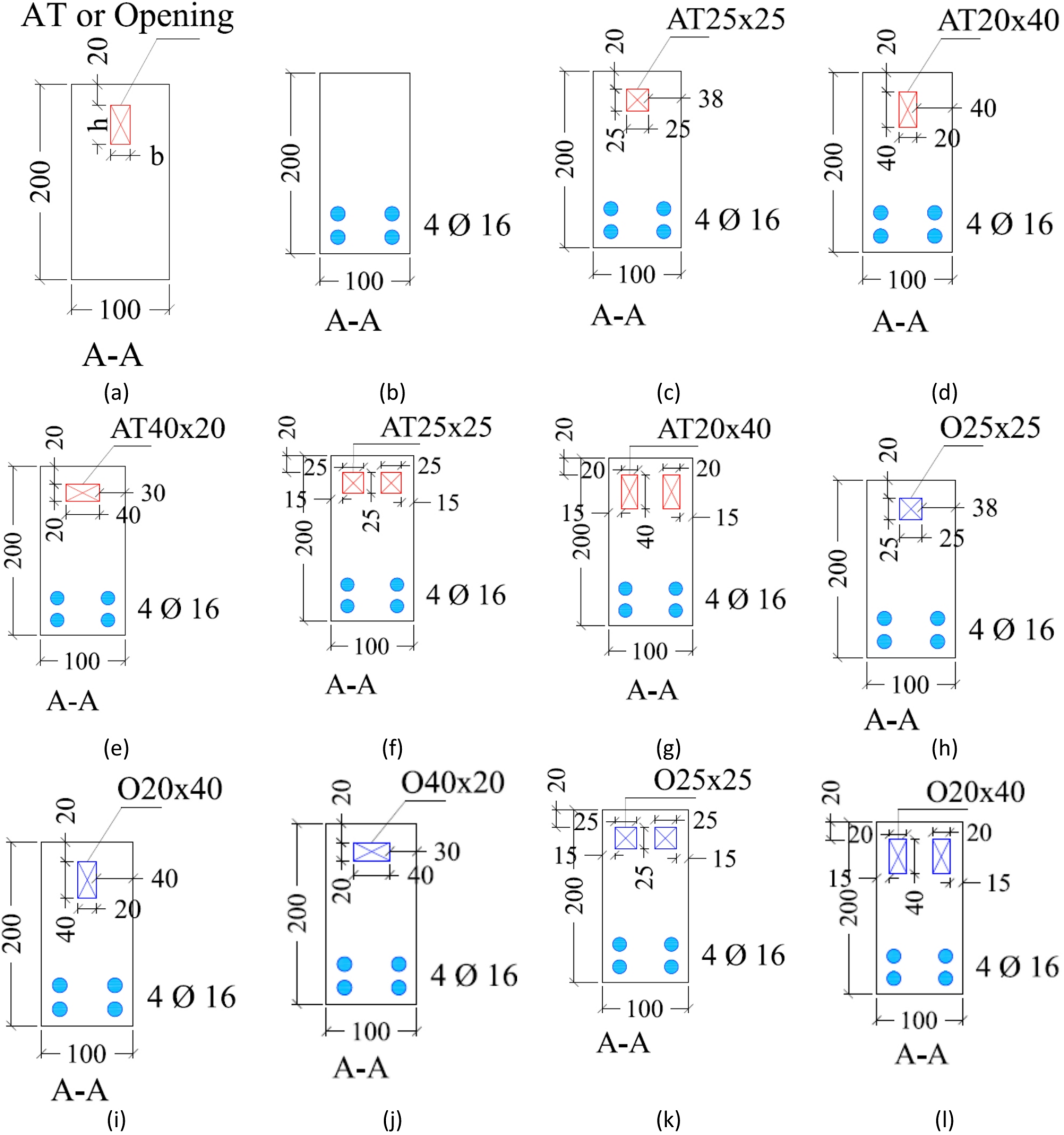
Beam section configurations at mid-span (mm).

The experimental program comprised eleven RC beam specimens with various internal configurations to investigate the influence of voids and embedded aluminum tubes on the structural performance of over-reinforced RC beams. Figures 3, 4 and Table 4 present the detailed specifications of all tested specimens. All beams shared identical geometric dimensions, with a width of 100 mm, an overall depth of 200 mm, and a total length of 1200 mm. Each beam was reinforced with four 16 mm diameter steel bars in the tension zone and two 16 mm diameter steel bars in the compression zone, limited to 350 mm at both ends of the beam. This study is focusing on performance of over-reinforced RC beams so these beams were reinforced with tension reinforcement ratio larger than balanced reinforcement ratio. The actual tension reinforcement ratio of presented beams was 0.0235 while balanced reinforcement ratio was 0.0220. The central region between the loading points was left unreinforced in compression and without shear reinforcement. Shear reinforcement consisted of 8 mm diameter stirrups spaced at 80 mm intervals, applied only within the 350 mm segments at both beam ends, as shown in Fig. 3.

**Table 4:** Beams matrix.

Beam	Opening geometry					Aluminum tube (AT) geometry					
	N	b (mm)	h (mm)	Ao (mm^2^)	α (%)	N	b (mm)	h (mm)	t (mm)	Aa (mm^2^)	λa (%)
B0	0.0	0.0	0.0	0.0	0.0	0.0	0.0	0.0	0.0	0.0	0.00
OI2.5 × 2.5	1.0	25.0	25.0	625.0	3.1	0.0	0.0	0.0	0.0	0.0	0.00
OI2 × 4	1.0	20.0	40.0	800.0	4.0	0.0	0.0	0.0	0.0	0.0	0.00
OI4 × 2	1.0	40.0	20.0	800.0	4.0	0.0	0.0	0.0	0.0	0.0	0.00
OII2.5 × 2.5	2.0	25.0	25.0	1250.0	6.3	0.0	0.0	0.0	0.0	0.0	0.00
OII2 × 4	2.0	20.0	40.0	1600.0	8.0	0.0	0.0	0.0	0.0	0.0	0.00
AI2.5 × 2.5	1.0	25.0	25.0	625.0	3.1	1.0	25.0	25.0	1.5	150.0	0.75
AI2 × 4	1.0	20.0	40.0	800.0	4.0	1.0	20.0	40.0	1.5	180.0	0.90
AI4 × 2	1.0	40.0	20.0	800.0	4.0	1.0	40.0	20.0	1.5	180.0	0.90
AII2.5 × 2.5	2.0	25.0	25.0	1250.0	6.3	2.0	25.0	25.0	1.5	300.0	1.50
AII2 × 4	2.0	20.0	40.0	1600.0	8.0	2.0	20.0	40.0	1.5	360.0	1.80

N, number; b, width; h, height; Ao, opening area; α, opening to section area ratio = Ao/Ac; t: AT thickness; Aa: AT area; λa: AT to section area ratio = Aa/Ac

The control specimen (B0) was solid, without any internal voids or embedded elements. The hollow beams were categorized based on the number and geometry of internal openings, with single-void specimens (OI2.5 × 2.5, OI2 × 4, OI4 × 2) containing one rectangular opening and double-void specimens (OII2.5 × 2.5, OII2 × 4) containing two. The area of the openings ranged from 625 mm^2^ to 1600 mm^2^, corresponding to an opening-to-section area ratio (α) between 3.1% and 8.0%. To further assess structural enhancement, a corresponding set of beams incorporated aluminum tubes (AT) with geometries matching the voids (AI2.5 × 2.5, AI2 × 4, AI4 × 2, AII2.5 × 2.5, AII2 × 4). The ATs had a uniform thickness of 1.5 mm, with cross-sectional areas ranging from 150 mm^2^ to 360 mm^2^, yielding AT-to-section area ratios (λa) from 0.75% to 1.80%.

### Casting and specimens’ preparation

**Fig. 5:**
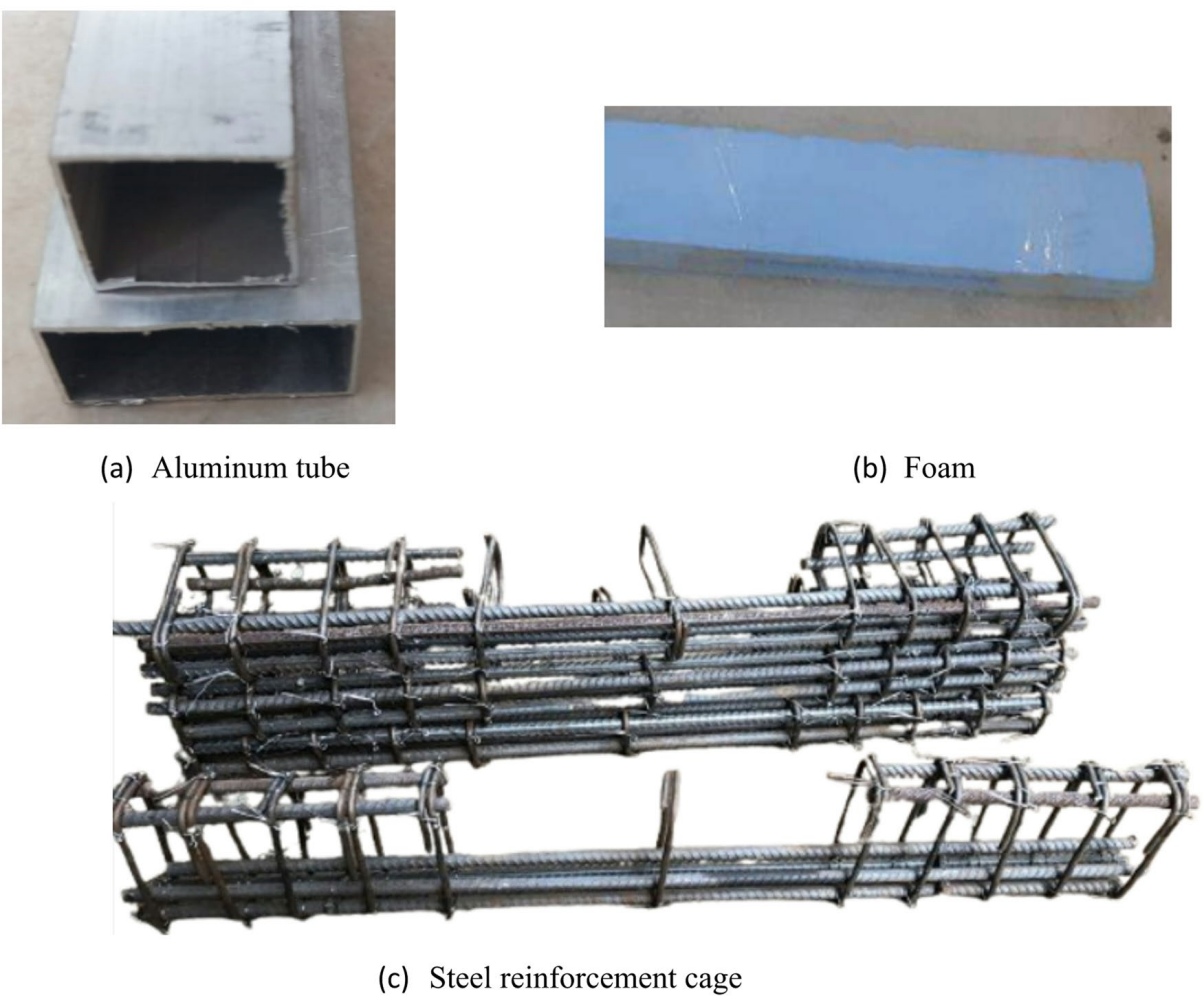
Materials used in specimen preparation.

The preparation of the eleven tested RC beams followed a systematic three-stage process to ensure consistency and accuracy across all specimens. The first stage involved the fabrication of aluminum tubes (ATs) according to the specified geometric dimensions, as illustrated in Fig. 5. These tubes were cut and shaped to fit precisely within the beam cross-section, ensuring proper alignment during embedding (Fig. 5a). Additionally, compressed foam (Fig. 5b) was used to form the voids according to the required dimensions.

The second stage consisted of assembling the reinforcement cages (Fig. 5c). Steel reinforcement was carefully prepared based on the design requirements, which included four 16 mm diameter bars for tension and two 16 mm diameter bars for compression, with appropriate stirrup arrangements at both ends. Special attention was given to securing the reinforcement in place, especially in beams containing internal voids or embedded ATs, to prevent displacement during casting.

The third stage involved the casting of normal concrete (NC). The concrete was mixed using a standard mix design and poured in a single continuous operation. Proper compaction was ensured using mechanical vibrators to eliminate entrapped air and achieve full consolidation around the reinforcement and any embedded aluminum elements. All beams, whether containing aluminum tubes or not, were cast using the same procedure to maintain uniformity across the test series. After casting, the specimens were cured under standard laboratory conditions for 28 days before testing.

### Testing device

**Fig. 6:**
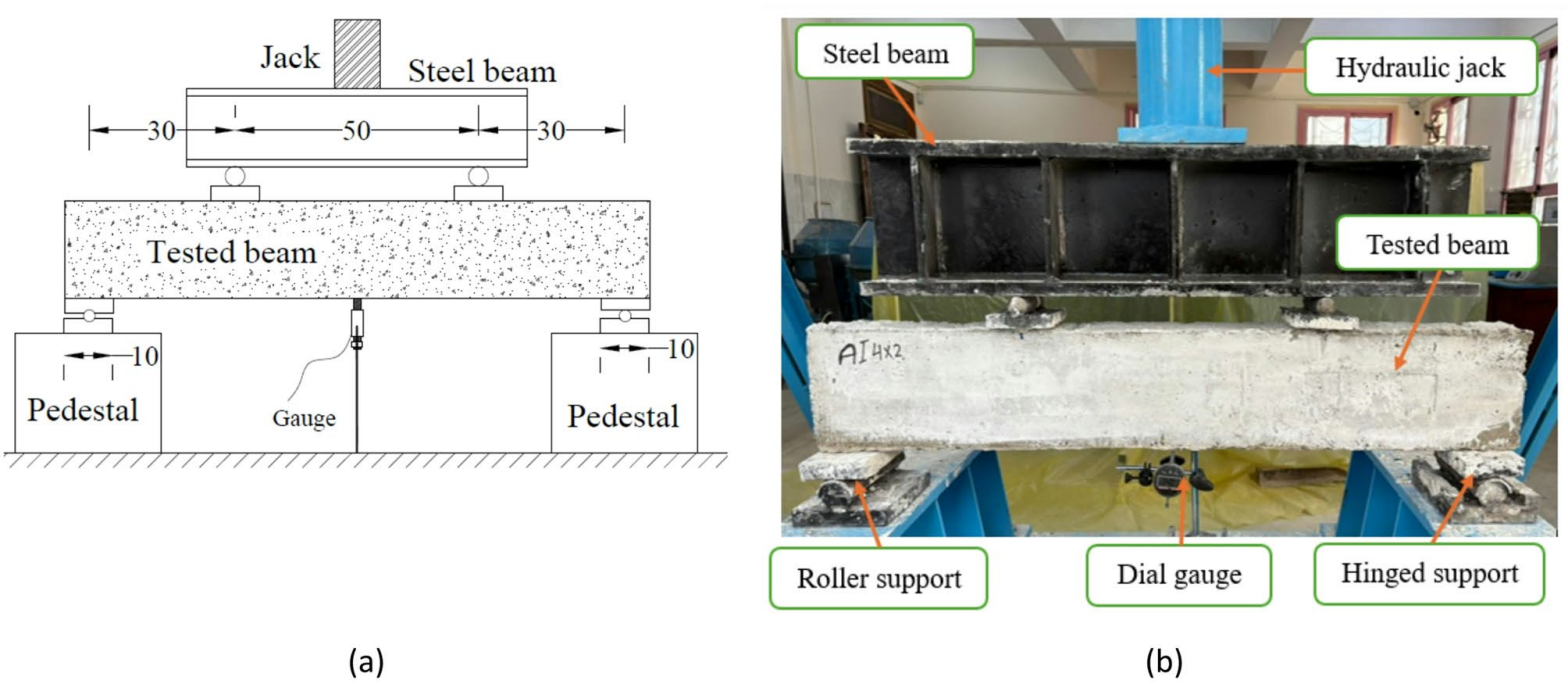
Test set up.

As shown in Fig. 6, all beams were tested using a four-point bending setup to evaluate their structural behavior. A 50-ton capacity hydraulic jack was mounted vertically at the center of a steel spreader beam to apply two equal concentrated loads, positioned symmetrically at 350 mm from the beam ends. This configuration created a constant moment region between the loading points and shear-dominated zones near the supports. The load was applied in a force-controlled manner, and the mid-span deflection was measured using a mechanical dial gauge placed directly beneath the beam’s center. Each beam was simply supported, with one end resting on a hinged support and the other on a roller support, allowing rotation and horizontal movement to minimize restraint-induced stresses. Load and deflection data were continuously recorded to capture the complete load–displacement response up to failure.

## Results and analysis

The experimental results of the tested RC beams, systematically categorized into seven distinct groups, are comprehensively summarized in Table 5. Each group was designed based on specific void characteristics—such as ratio, number, and orientation—and reinforcement strategies, including the presence or absence of embedded aluminum tubes. The evaluation encompasses detailed analyses of failure modes, load–deflection (P–δ) responses, and the overall structural behavior of the beams. This categorization facilitates a clear comparison of the impact of void configurations and internal reinforcement on the flexural performance and ductility of RC beams.

**Table 5:** Results of tested beams.

Variable	Group	Beam	α (%)	λa (%)	P_u_ (kN)	Change (%)	δu (mm)	Change (%)	k (kN/mm)	Change (%)	EA (kN.mm)	Change (%)	Ductility index (DI)	Change (%)
Void ratio	A	B0	0	0	123.1	0.00	5.02	0.00	45	0.00	290	0.00	1.26	0.00
		OI2.5 × 2.5	3.1	0	100.1	−18.68	3.5	−30.28	38.4	−14.67	226	−22.07	1.30	3.17
		OI2 × 4	4	0	89.6	−27.21	3.03	−39.64	38.9	−13.56	183	−36.90	1.40	11.10
		OII2.5 × 2.5	6.2	0	87.77	−28.70	3.55	−29.28	38.1	−15.33	173	−40.34	1.71	35.71
		OII2 × 4	8	0	78.23	−36.45	3.8	−24.30	25.2	−44.00	160	−44.83	1.78	41.27
Voids number	B	OI2.5 × 2.5	3.1	0	100.1	0.00	3.5	0.00	38.4	0.00	226	0.00	1.30	0.00
		OII2.5 × 2.5	6.2	0	87.77	−12.32	3.55	1.43	38.1	−0.78	173	−23.45	1.71	31.54
		OI2 × 4	4	0	89.6	0.00	3.03	0.00	38.9	0.00	183	0.00	1.40	7.70
		OII2 × 4	8	0	78.23	−12.69	3.8	25.41	25.2	−35.22	160	−12.57	1.78	36.92
Void orientation	C	OI2 × 4	4	0	89.6	0.00	3.03	0.00	38.9	0.00	183	0.00	1.40	0.00
		OI4 × 2	4	0	85.3	−4.80	3.02	−0.33	45	15.68	175	−4.37	1.37	−2.114
Void reinforcing by ATs	D	OI2.5 × 2.5	3.1	0	100.1	0.00	3.5	0.00	38.4	0.00	226	0.00	1.30	0.00
		AI2.5 × 2.5	3.1	0.75	157.3	57.14	5.22	49.14	45.2	17.71	512	126.55	1.30	0.00
		OI2 × 4	4	0	89.6	0.00	3.03	0.00	38.9	0.00	183	0.00	1.40	0.00
		AI2 × 4	4	0.9	167.2	86.61	4.9	61.72	44.9	15.42	484	164.48	1.27	−9.30
		OI4 × 2	4	0	85.3	0.00	3.02	0.00	45	0.00	175	0.00	1.37	0.00
		AI4 × 2	4	0.9	185.2	117.12	5.2	72.19	45.23	0.51	575	228.57	1.30	−5.20
		OII2.5 × 2.5	6.2	0	87.77	0.00	3.55	0.00	38.1	0.00	173	0.00	1.71	0.00
		AII2.5 × 2.5	6.2	1.5	195.3	122.51	5.04	41.97	44.2	16.01	561	224.28	1.27	−25.70
		OII2 × 4	8	0	78.23	0.00	3.8	0.00	25.2	0.00	160	0.00	1.78	0.00
		AII2 × 4	8	1.8	184.2	135.46	5.15	35.53	45.4	80.16	582	263.75	1.30	−27.0
AT area ratio	E	B0	0	0	123.1	0.00	5.02	0.00	45	0.00	290	0.00	1.26	0.00
		AI2.5 × 2.5	3.1	0.75	157.3	27.78	5.22	3.98	45.2	0.44	512	76.55	1.30	3.17
		AI2 × 4	4	0.9	167.2	35.82	4.9	−2.39	44.9	−0.22	484	66.90	1.27	0.80
		AI4 × 2	4	0.9	185.2	50.45	5.2	3.59	45.23	0.51	575	98.28	1.30	3.17
		AII2.5 × 2.5	6.2	1.5	195.3	58.65	5.04	0.40	44.2	−1.78	561	93.45	1.27	0.80
		AII2 × 4	8	1.8	184.2	49.63	5.15	2.59	45.4	0.89	582	100.69	1.30	3.17
Number of voids reinforced with ATs	B*	AI2.5 × 2.5	3.1	0.75	157.3	0.00	5.22	0.00	45.2	0.00	512	0.00	1.30	0.00
		AII2.5 × 2.5	6.2	1.5	195.3	24.16	5.04	−3.45	44.2	−2.21	561	9.57	1.27	−2.30
		AI2 × 4	4	0.9	167.2	0.00	4.9	0.00	44.9	0.00	484	0.00	1.27	−2.30
		AII2 × 4	8	1.8	184.2	10.17	5.15	5.10	45.4	1.11	582	20.25	1.30	0.00
Orientation of voids reinforced with ATs	C*	AI2 × 4	4	0.9	167.2	0.00	4.9	0.00	44.9	0.00	484	0.00	1.27	0.00
		AI4 × 2	4	0.9	185.2	10.77	5.2	6.12	45.23	0.73	575	18.80	1.30	2.36

### Failure patterns

**Fig. 7:**
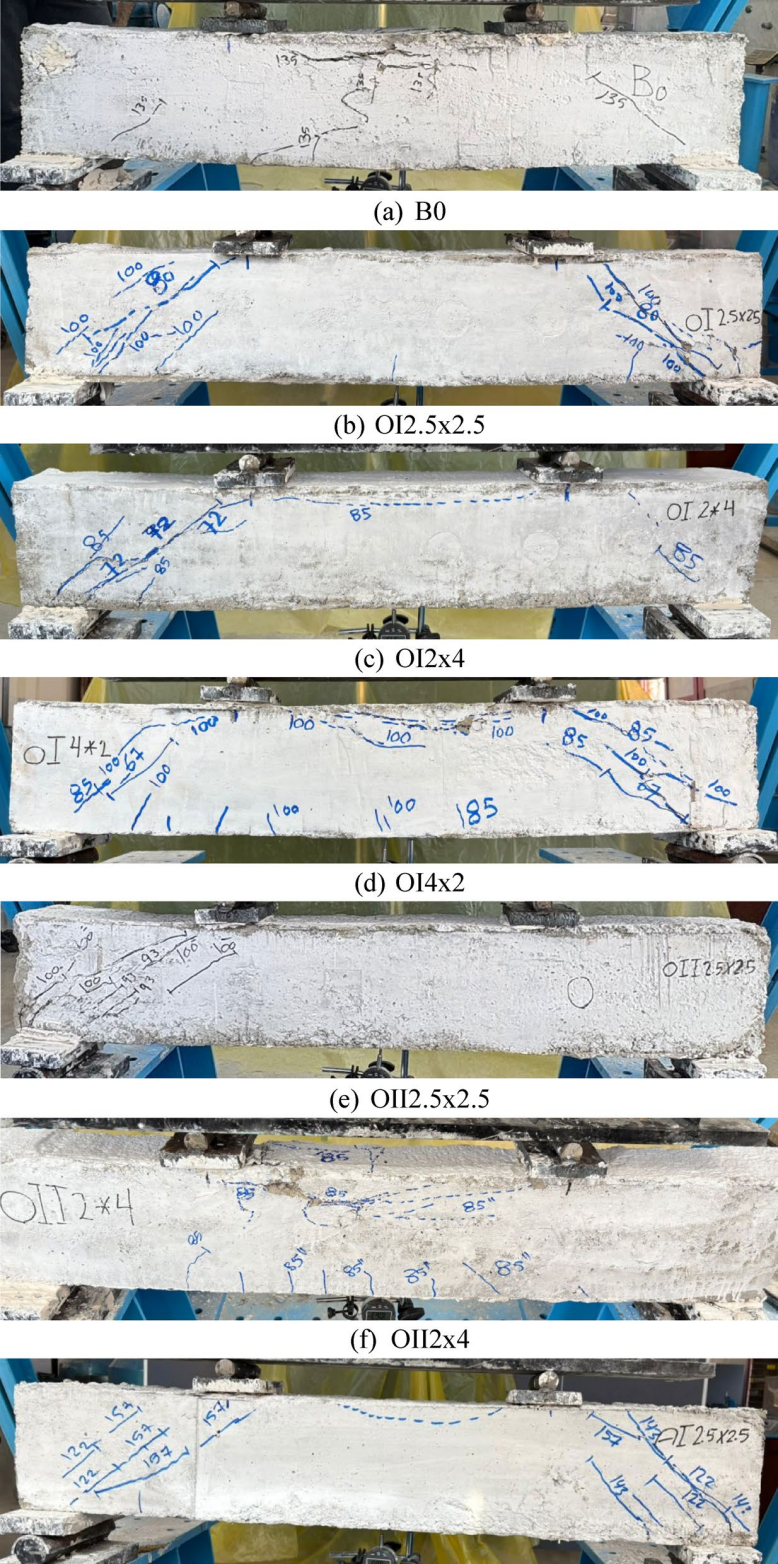

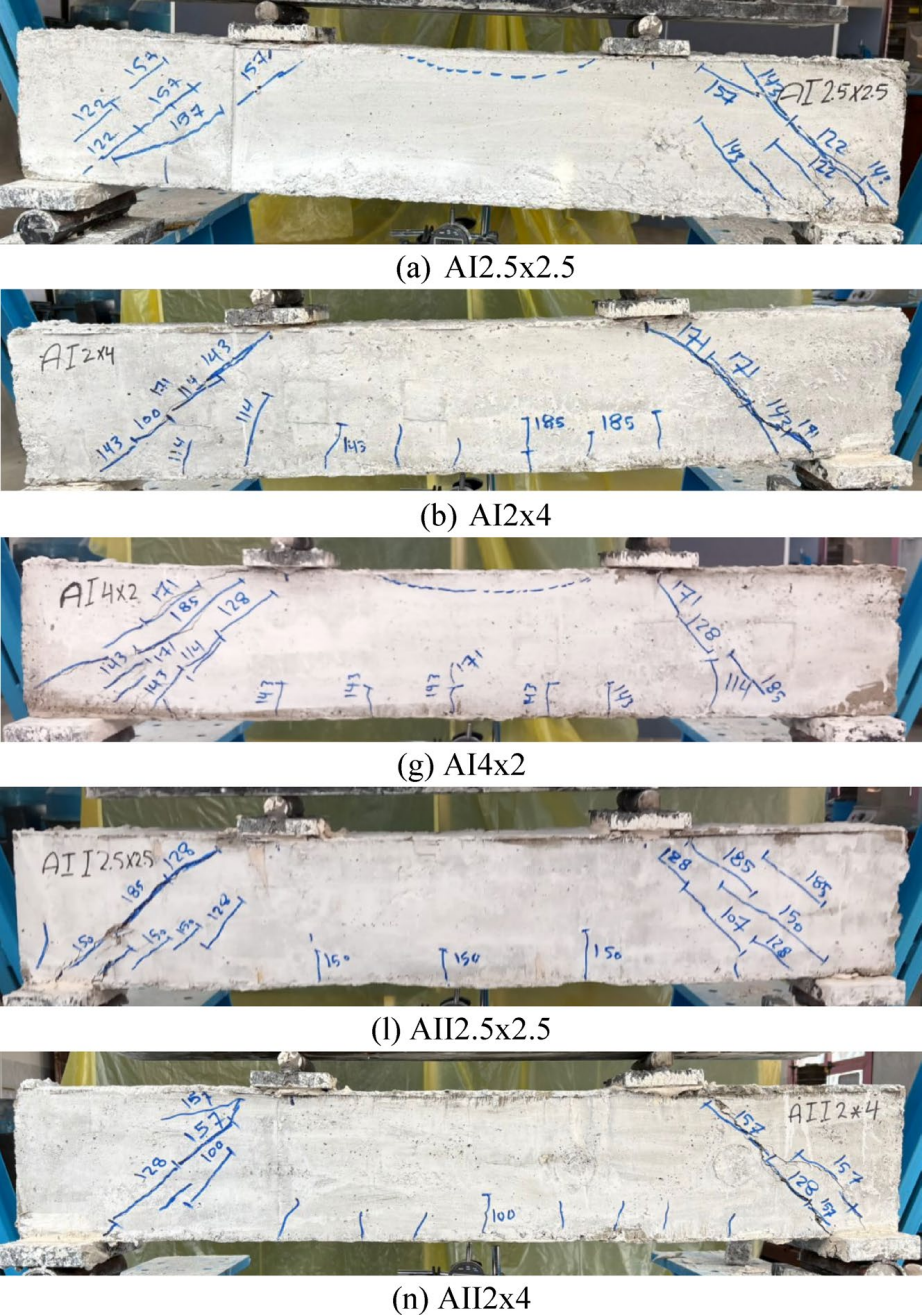
Collapses and cracks of all beams.

Figure 7 shows failures of the tested beams. All beams showed shear cracks in shear spans between loading point and support. With starting loading, first shear crack occurred. With increase of loading, this crack increase in the length and width as well as other shear cracks appeared. The shear cracks lengthened from the loading point to the support. Due to use of high reinforcement in the tension side of the beams, almost beams showed concrete crushing in the compression zone at the middle part of the beams including unreinforced voids (Figs. 7a, c, d, f, g). When aluminum tubes were used around these voids, the concrete crushing in the compression zone did not appear although these beams resisted loads more than those including unreinforced voids (Figs. 7 h, l, n). The voids caused a decrease in the concrete area in the compression zone, and after shear cracks developed, the concrete crushing at the openings occurred. This behavior improved when the voids were surrounded by aluminum tubes, which bore the compressive load and prevented the concrete crushing.

### Load versus deflection

**Fig. 8:**
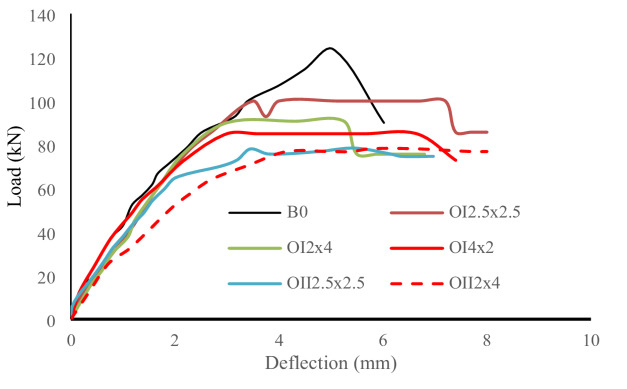
Effect of Void ratio, number and orientation on the P-δ relationships of group A, B and C.

**Fig. 9:**
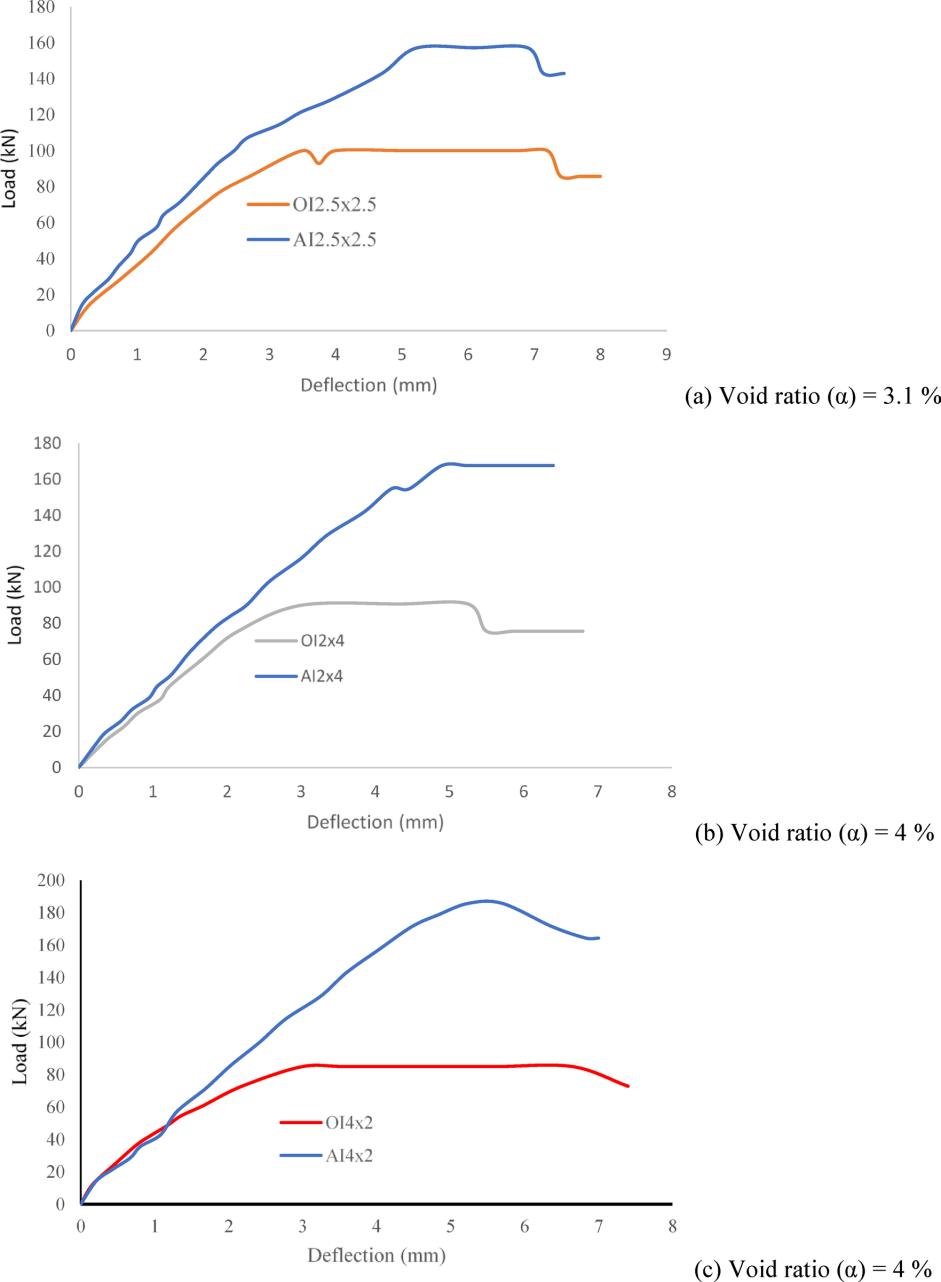

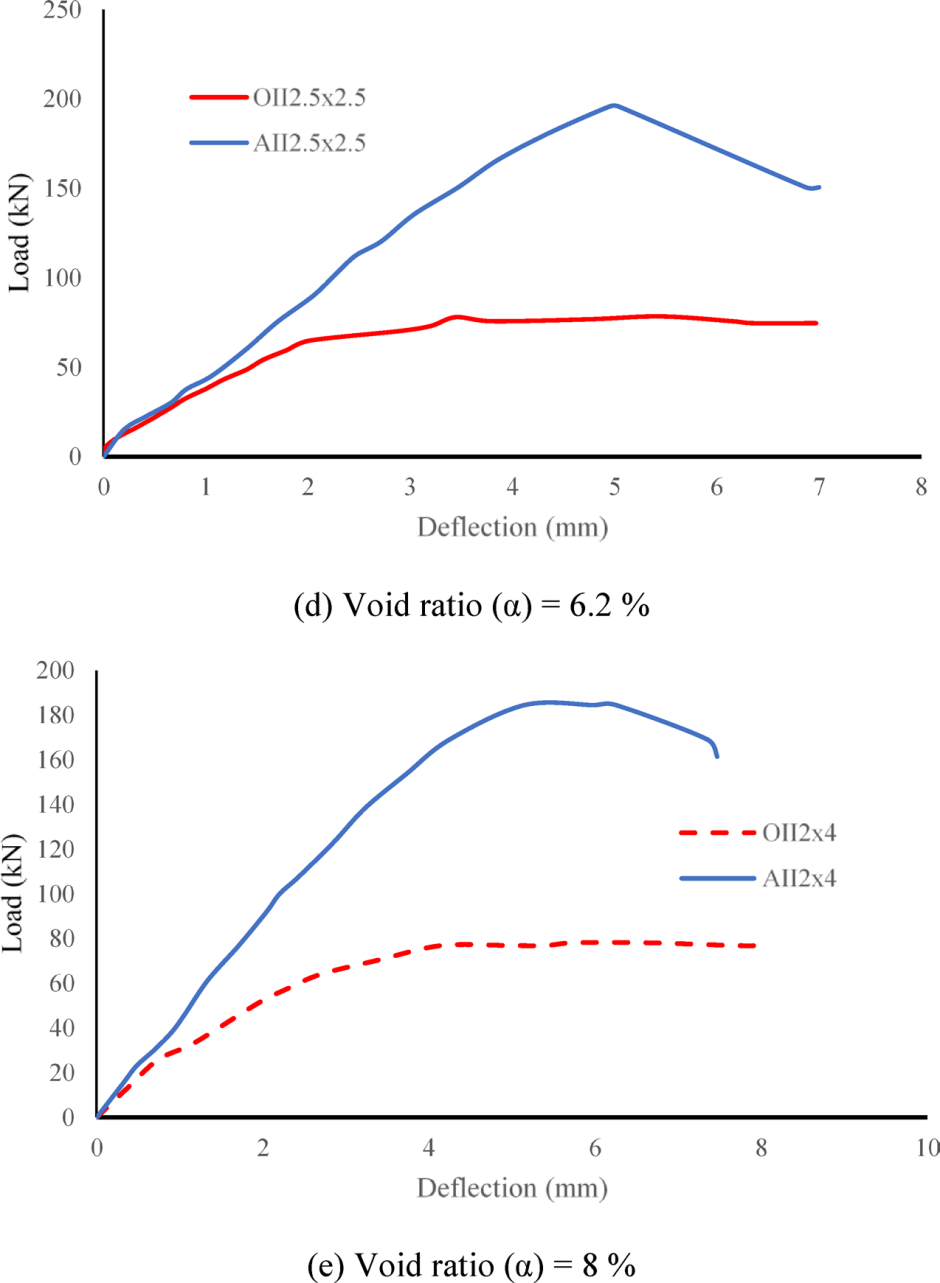
Effect of Void reinforcing by ATs on the P-δ relationships of group D.

**Fig. 10:**
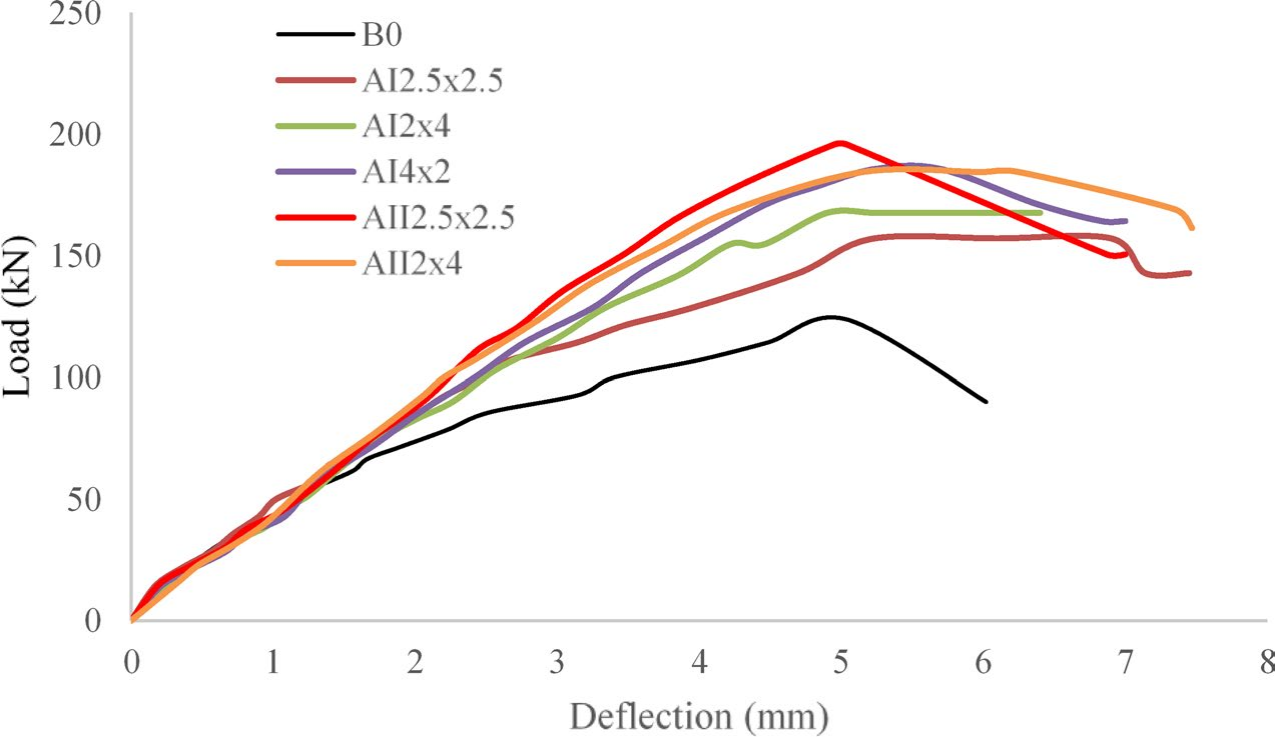
Effect of AT area ratio on the P-δ relationships of group E.

The results of all the tested beams under monotonic loading are given in Figs. 8, 9, 10 as load–deflection (P–δ) responses respectively. Commonly, the curves show three phases: (i) an initial linear elastic stage until first cracking, (ii) a nonlinear post-cracking phase subject to stiffness degradation and crack growth, and lastly (iii) a post-peak softening characteristic of stiffness loss with progressive failure. Variations in these stages are indicative of the effects of void configuration and aluminum tube (AT) reinforcement. Beams from Groups A–C are compared to analyze the influence of void ratio, number, and orientation (Fig. 8). The stiffness and ultimate load (P_u_) performance was identified for the solid control beam (B0), while all voided beams led to a reduction of overall performance. The rational deduction confirmed the negative influence of the section discontinuity on P_u_ and energy absorption with increasing void ratio and number. Among beams with similar void ratios, the vertically oriented void (OI2 × 4) exhibited better performance than the horizontally oriented one (OI4 × 2). This indicates a stronger coupling effect associated with aligned openings, which results in less disturbance to the stress flow. Similar trends have been reported in previous studies^16^. Figure 9(a–e) show how Group D beams act, with a focus on the part that embedded aluminum tubes play. For all void configuration (3.1%–8%), AT-reinforced beams were always stiffer, had higher P_u_, and were more ductile than beams without reinforcement. The change was most noticeable in the post-cracking and post-peak, when aluminum tubes helped spread out the stress and put off failure. This behavior is consistent with literature on internal confinement and composite action, which indicates that embedded metallic components improve load transfer and energy absorption capabilities^31,32^.

Figure 10 shows the influence of AT area ratio (Group E). While the initial elastic stiffness was only marginally affected, increasing the AT ratio significantly improved post-cracking behavior, ultimate strength, and deformation capacity. Beams with higher aluminum content exhibited more stable post-peak responses and greater energy dissipation, demonstrating the effectiveness of ATs in compensating for the reduced concrete area. These results are consistent with previous research on steel or composite reinforcement in voided members, where increased reinforcement ratio enhances ductility and delays structural degradation. Overall, the results confirm that although compression-zone voids adversely affect structural performance, the incorporation of aluminum tubes not only mitigates these effects but can also restore or exceed the behavior of solid beams, particularly in terms of ductility and energy absorption.

### Overall characteristics

From P-δ curve of each beam, ultimate load capacity (${P}_{u}$) was obtained at the peak point. Deflection corresponding to the ${P}_{u}$ (${\delta}_{u}$) also observed. Elastic stiffness (k), which was equal to the slope of the curve’s initial linear part of the P-δ curve, was estimated. Energy absorbed (EA), which is defined as the area under the P-δ curve up to the peak point, was estimated. Additionally, the ductility index (DI) was used to assess the ductility property of the tested beams. The ductility index (DI) was calculated^33^ by Eq. (1).

1 $$DI={\delta}_{u}/{\delta}_{y}$$

where δu is the axial displacement corresponding to the ultimate strength, and δy is taken as δ_75%_, where δ_75%_ is the axial displacement before failure corresponding to 75% of the ultimate load. These parameters were acquired for each group and are shown in Table 5.

#### Effect of void ratio (group A)

**Fig. 11:**
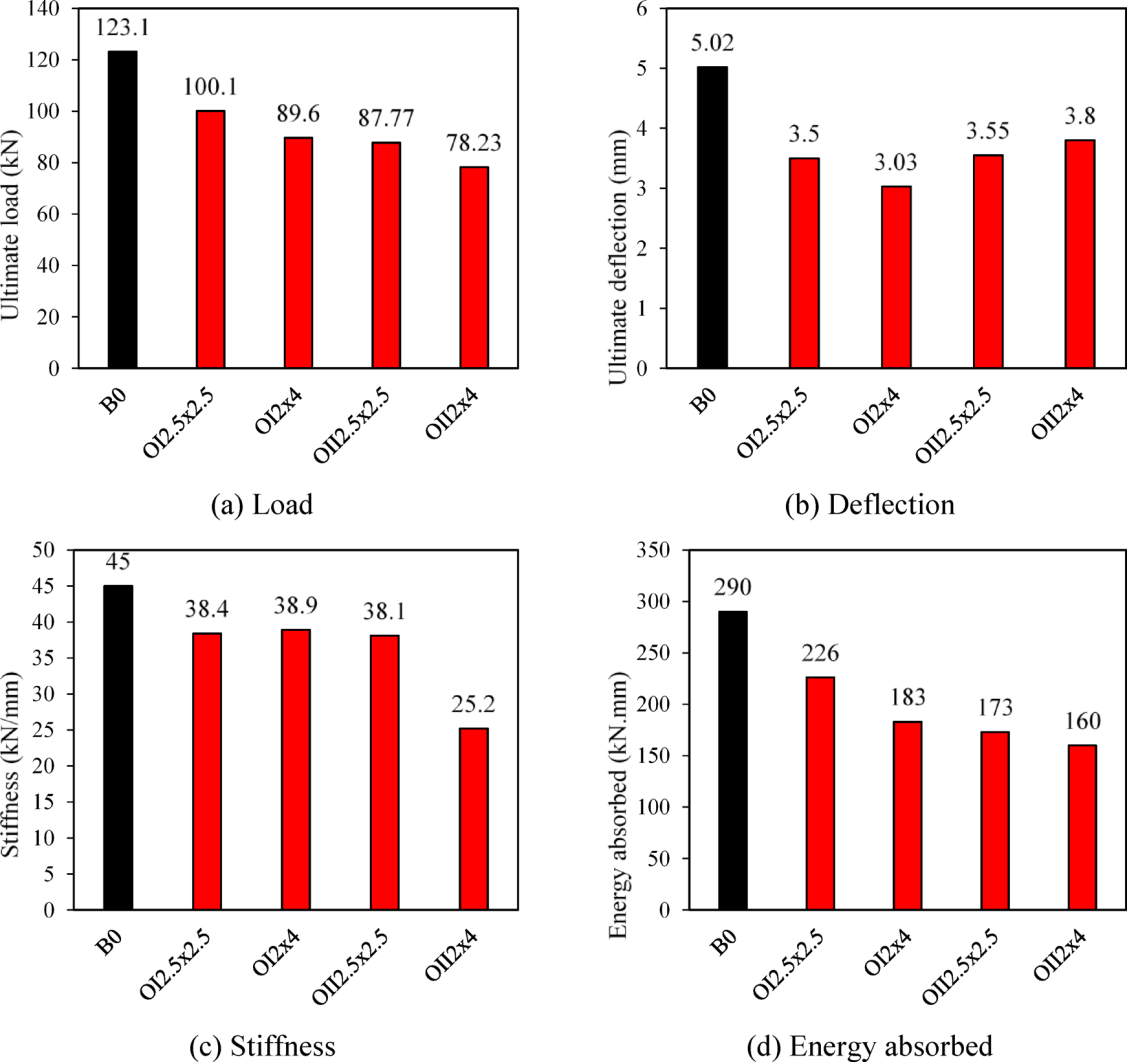
Effect of void ratio on performance of tested beams.

Group A examines the influence of void ratio on the flexural behavior of RC beams without internal reinforcement (Fig. 11). The group consists of one solid reference beam (B0) and four hollow beams with compression-zone void ratios ranging from 3.1% to 8%. The control beam (B0) exhibited the best overall performance (${P}_{u}$= 123.1 kN, ${\delta}_{u}$ = 5.02 mm, k = 45 kN/mm, EA = 290 kN·mm). Introducing voids led to a consistent degradation in structural response. Even a small void ratio of 3.1% caused noticeable reductions in ultimate load (− 18.7%) and energy absorption (− 22.1%). As the void ratio increased to 4%, 6.2%, and 8%, the reduction in ${P}_{u}$ became more pronounced (up to − 36.5%), accompanied by significant losses in stiffness (up to − 44%) and energy absorption (up to − 44.8%). This trend reflects the progressive weakening of the compression zone and disruption of the internal stress flow due to section discontinuity. Similar observations have been reported in studies on RC beams with openings, where increasing void size leads to reduced load capacity and earlier crushing of the compression zone^34–36^. Void geometry also played a critical role. Beams with deeper, vertically oriented voids (e.g., 2 × 4 cm) exhibited greater strength and stiffness reductions compared to more compact, square openings (2.5 × 2.5 cm), even at comparable void ratios. This behavior can be attributed to higher stress concentration and reduced effective compression area in elongated void configurations, consistent with findings in the literature on web openings and sectional discontinuities^2^.

In terms of deformation, all hollow beams showed reduced ultimate deflection (by approximately 30–40%), indicating diminished ductility. However, the ductility index (DI) exhibited an increasing trend with higher void ratios, rising from 1.26 for the control beam to 1.78 (an increase of 41.27%) for the beam with the largest void ratio. This apparent contradiction suggests that although voided beams fail at lower deformation levels, they experience a more gradual post-yield response relative to their reduced yield capacity. Slightly higher deflection and more favorable ductility behavior were observed in beams with square voids due to improved stress distribution. Overall, increasing void ratio and unfavorable geometry promote premature cracking, reduce load-carrying capacity, and shift the response toward a more brittle yet relatively more deformable post-yield behavior.

#### Effect of voids number (group B)

**Fig. 12:**
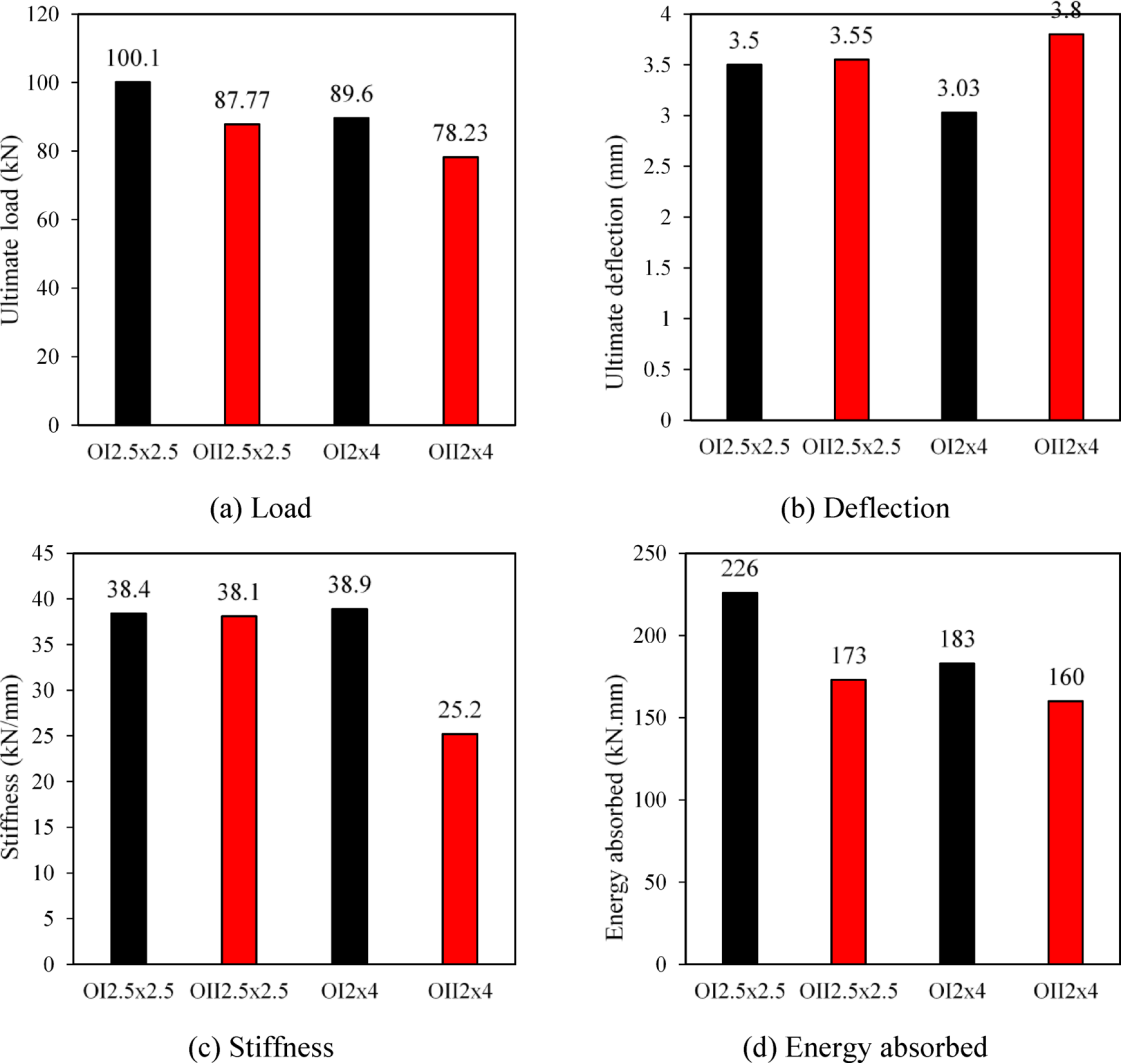
Effect of void number on performance of tested beams.

Group B investigates the effect of void number and geometry on the performance of over-reinforced RC beams without internal reinforcement, where all voids are positioned in the compression zone (Fig. 12). The configurations include single and double voids of identical shapes: square (OI2.5 × 2.5 and OII2.5 × 2.5 with 3.1% and 6.2%) and rectangular (OI2 × 4 and OII2 × 4 with 4% and 8%), allowing a direct evaluation of the influence of void number. The results indicate that increasing the number of voids consistently degrades structural performance. For square openings, doubling the voids reduced the ${P}_{u}$ by 12.3% and EA by 23.5%, with minimal impact on stiffness. In contrast, rectangular voids showed more severe deterioration, with reductions of 12.7% in ${P}_{u}$, 35.2% in stiffness, and 12.6% in EA. This behavior is attributed to intensified stress concentrations and weakening of the compression zone, which limit stress redistribution. The effect is more pronounced for rectangular openings due to their greater depth and disruption of load paths.

**Fig. 13:**
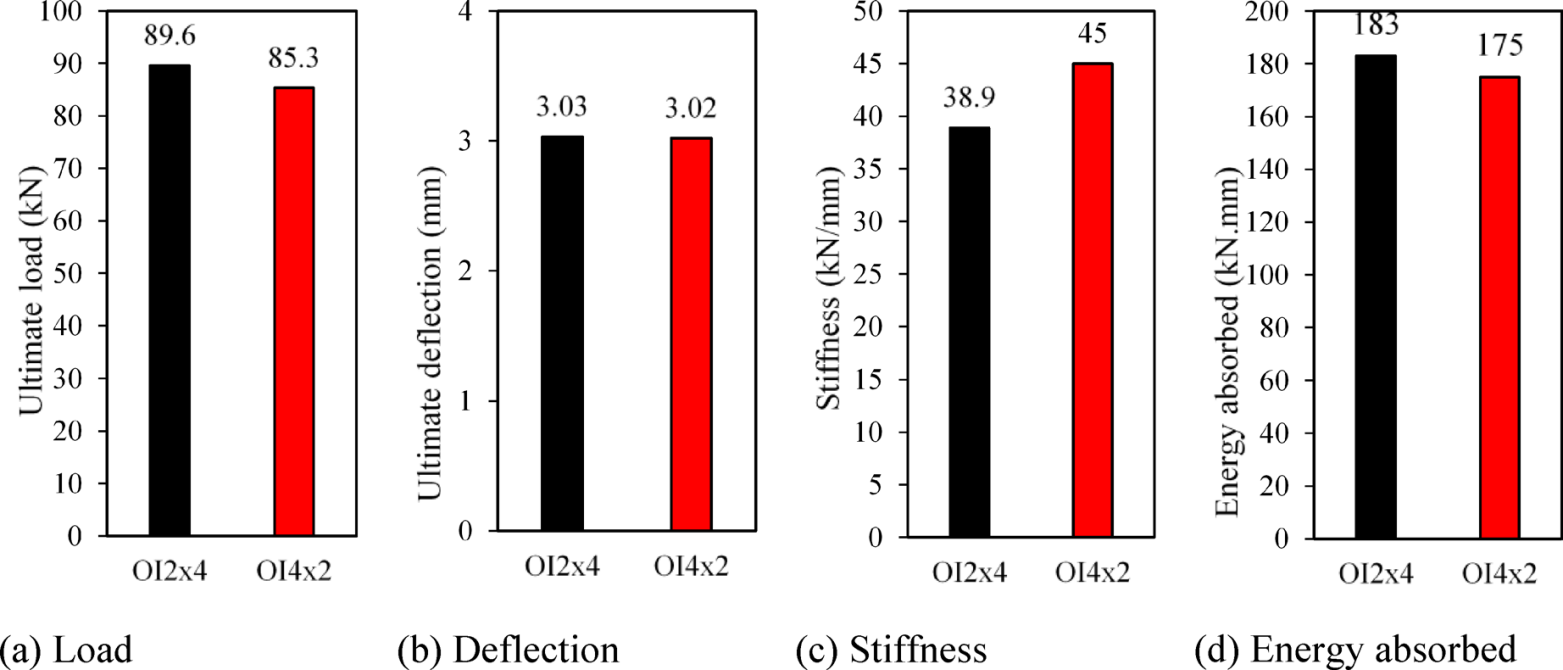
Effect of void orientation on performance of tested beams.

In terms of deformation, square voids caused only minor increases in ultimate deflection, whereas double rectangular voids increased deflection by approximately 25%. However, this increase reflects premature cracking and stiffness loss rather than improved ductility. This is confirmed by the reduction in energy absorption and brittle failure characteristics. Although the ductility index (DI) increased with void number—by 31.54% for square voids and 36.92% for rectangular voids—this apparent improvement is misleading. It results from early cracking and extended deformation before failure, not from enhanced energy dissipation capacity. Overall, increasing void number significantly compromises beam performance, particularly for high-aspect-ratio openings, due to accelerated crack propagation, reduced stiffness, and diminished energy absorption capacity^37,38^ (Fig. 13).

#### Effect of void orientation (group C)

**Fig. 14:**
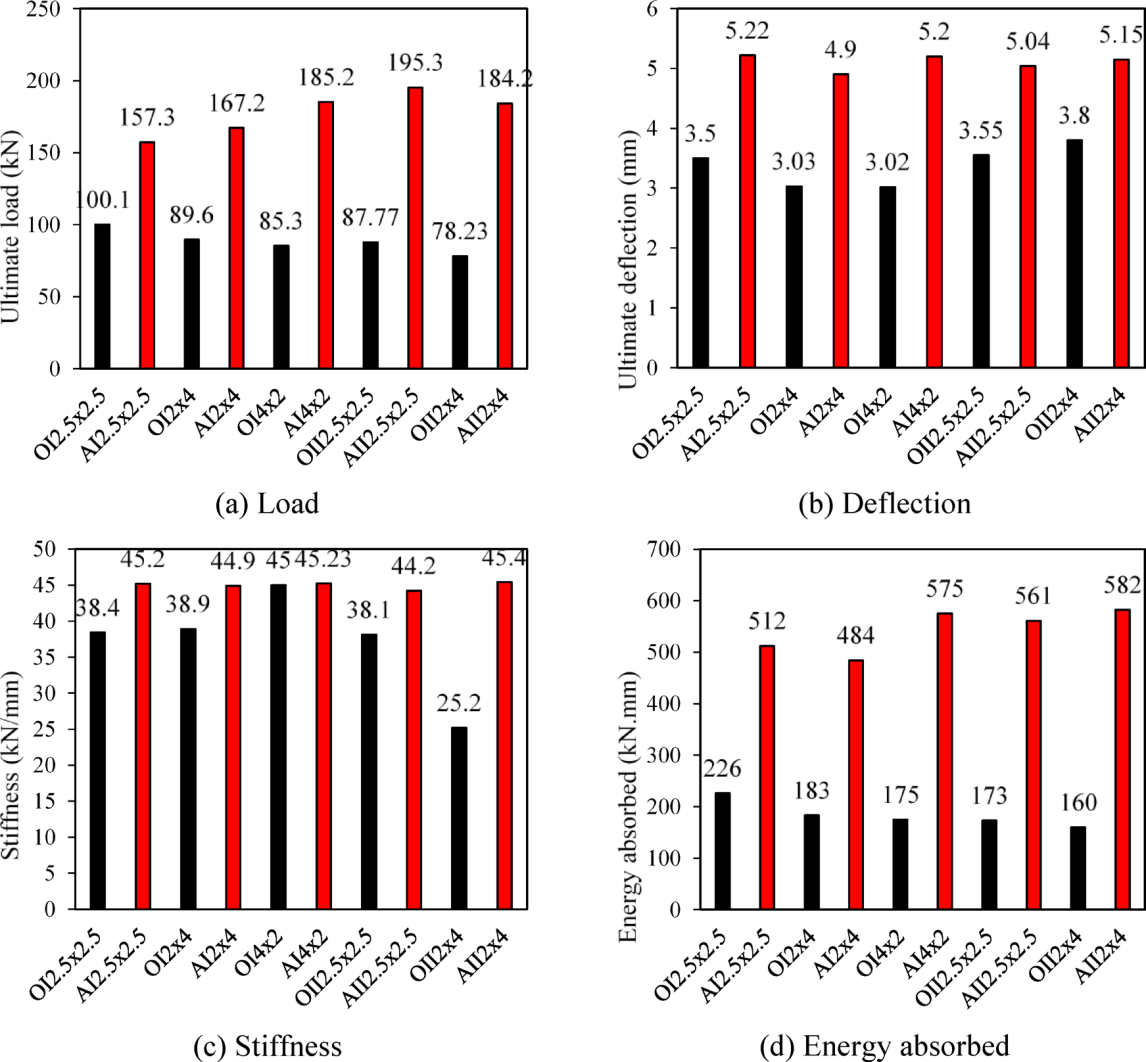
Effect of void reinforcement using ATs on performance of tested beams.

Two configurations were considered: vertically oriented voids (OI2 × 4) and horizontally oriented voids (OI4 × 2), both located in the compression zone to examines the influence of void orientation, as shown in Fig. 14. The results indicate that void orientation has a noticeable but moderate effect on structural performance. The beam with horizontal voids (OI4 × 2) exhibited a slightly lower ${P}_{u}$ (− 4.8%) compared to the vertically oriented beam (OI2 × 4), while the ${\delta}_{u}$ remained nearly identical (≈3.02–3.03 mm). In contrast, the horizontal configuration showed a significant increase in initial stiffness (+ 15.7%) and a minor reduction in energy absorption (≈ − 4.4%). In terms of ductility, only a marginal variation was observed. The ductility index decreased slightly from 1.40 for the vertically oriented voids to 1.37 for the horizontal configuration (− 2.11%), indicating that void orientation has a limited influence on post-yield deformation capacity. These differences are attributed to the interaction between void orientation and the compressive stress field. The vertical stress flow is less disrupted by horizontal voids, resulting in improved stiffness, whereas vertical voids preserve a more continuous compression path, contributing to slightly higher load capacity.

#### Effect of void reinforcing by ATs (group D)

Group D (Fig. 14) investigates the effect of embedding aluminum tubes (ATs) within voided RC beams by comparing each strengthened specimen with a geometrically identical unreinforced counterpart. The AT-to-section area ratio (λₐ) varies from 0.75% to 1.8%, covering single and multi-void configurations with different geometrical orientations. Overall, the incorporation of ATs markedly improves structural performance, particularly in terms of ${P}_{u}$ and EA, although ductility response is not always enhanced. For single-void beams, significant gains are observed: the AI2.5 × 2.5 specimen achieves a 57% increase in ${P}_{u}$ with more than 100% improvement in EA, while AI2 × 4 exhibits an 86.6% strength increase and a 164% rise in EA. The most efficient configuration is AI4 × 2, which records a 117% increase in ${P}_{u}$ and a 229% enhancement in EA, indicating strong sensitivity to void orientation.

For multi-void configurations, the strengthening effect becomes even more pronounced. AII2.5 × 2.5 shows a 122.5% increase in ${P}_{u}$ and over 220% improvement in EA, while AII2 × 4 provides the highest energy enhancement, reaching approximately 264% alongside a 135% strength increase. These results confirm the strong effectiveness of AT reinforcement in mitigating the adverse effects of multiple voids. However, ductility trends reveal a more complex behavior. While single-void reinforced beams generally maintain or slightly reduce ductility (DI changes around 0 to − 9.3%), multi-void strengthened beams show a noticeable reduction in ductility index (up to − 25.7% and − 27%), suggesting a stiffer but less deformable response due to increased confinement and composite action.

The significant improvement in the structural performance of the aluminum tube (AT)-reinforced beams can be attributed to a combination of confinement effect, composite interaction, and delayed instability of the embedded tubes, which collectively enhance the behavior of the compression zone. From a mechanical perspective, the embedded aluminum tubes act as longitudinal stiff inclusions within the compression region, partially replacing the crushed concrete core. Due to their relatively high axial stiffness compared to cracked concrete, the ATs participate in carrying compressive stresses after cracking occurs. This leads to a composite action between the surrounding concrete and the aluminum tubes, where load transfer is achieved through bond interaction and mechanical interlock at the concrete–aluminum interface. As a result, the compression block becomes more stable and less prone to localized crushing.

In addition, the presence of ATs provides a confinement-like effect to the surrounding concrete. The tubular geometry restricts lateral dilation of the adjacent concrete under compression, which delays the propagation of micro-cracks and enhances the post-peak response. This effect is particularly pronounced in beams with higher AT area ratios, where a larger portion of the compression zone is internally supported. Moreover, although aluminum tubes are slender elements, their embedment within concrete significantly reduces the risk of local buckling. The surrounding concrete provides continuous lateral restraint along the tube length, effectively increasing the critical buckling capacity of the tubes. This confinement delays instability and allows the aluminum tubes to sustain higher compressive stresses beyond initial cracking of concrete.

##### Improvement coefficient

Group D composed of five pairs of the beams. Any pair consisted of one included unreinforced void and one included void reinforced with ATs. For example, first pair consisted of two beams OI2.5 × 2.5 and AI2.5 × 2.5. Improvement coefficient (β) was estimated by following equation:

2 $$\upbeta = \frac{{P}_{u}}{{P}_{u0}}$$

where ${P}_{u}$ is ultimate load of the beam reinforced with AT around the void. ${P}_{uo}$ is ultimate load of the unreinforced beam with AT.

**Fig. 15:**
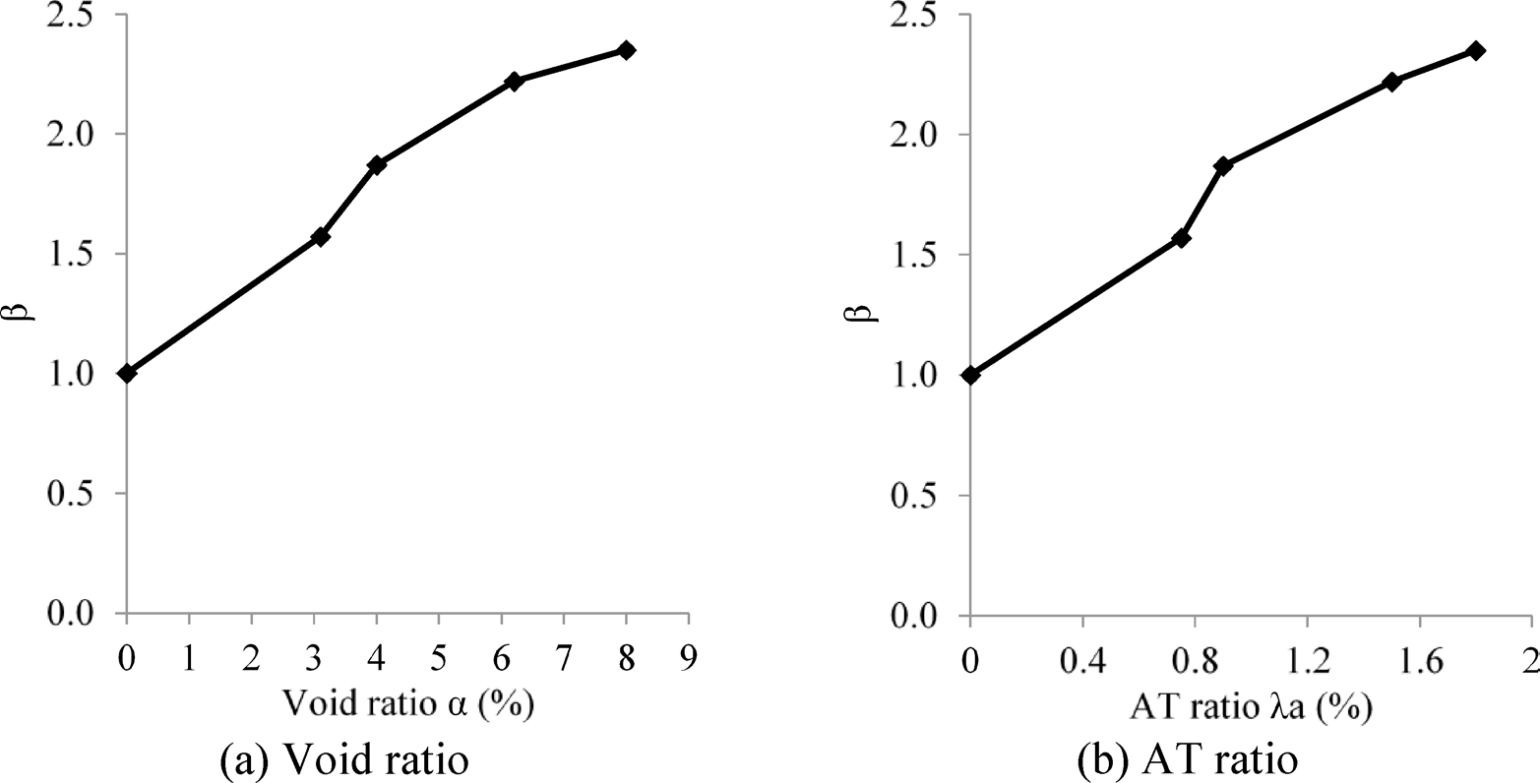
Improvement coefficient (β) versus void ratio and AT ratio.

Improvement coefficient (β) of the first pair was 1.57. Each pair had a same void ratio (α). The relationship between the β and α is drawn in Fig. 15a while the relationship between the β and AT ratio (λa) is drawn in Fig. 15b.

#### Effect of AT area ratio (group E)

**Fig. 16:**
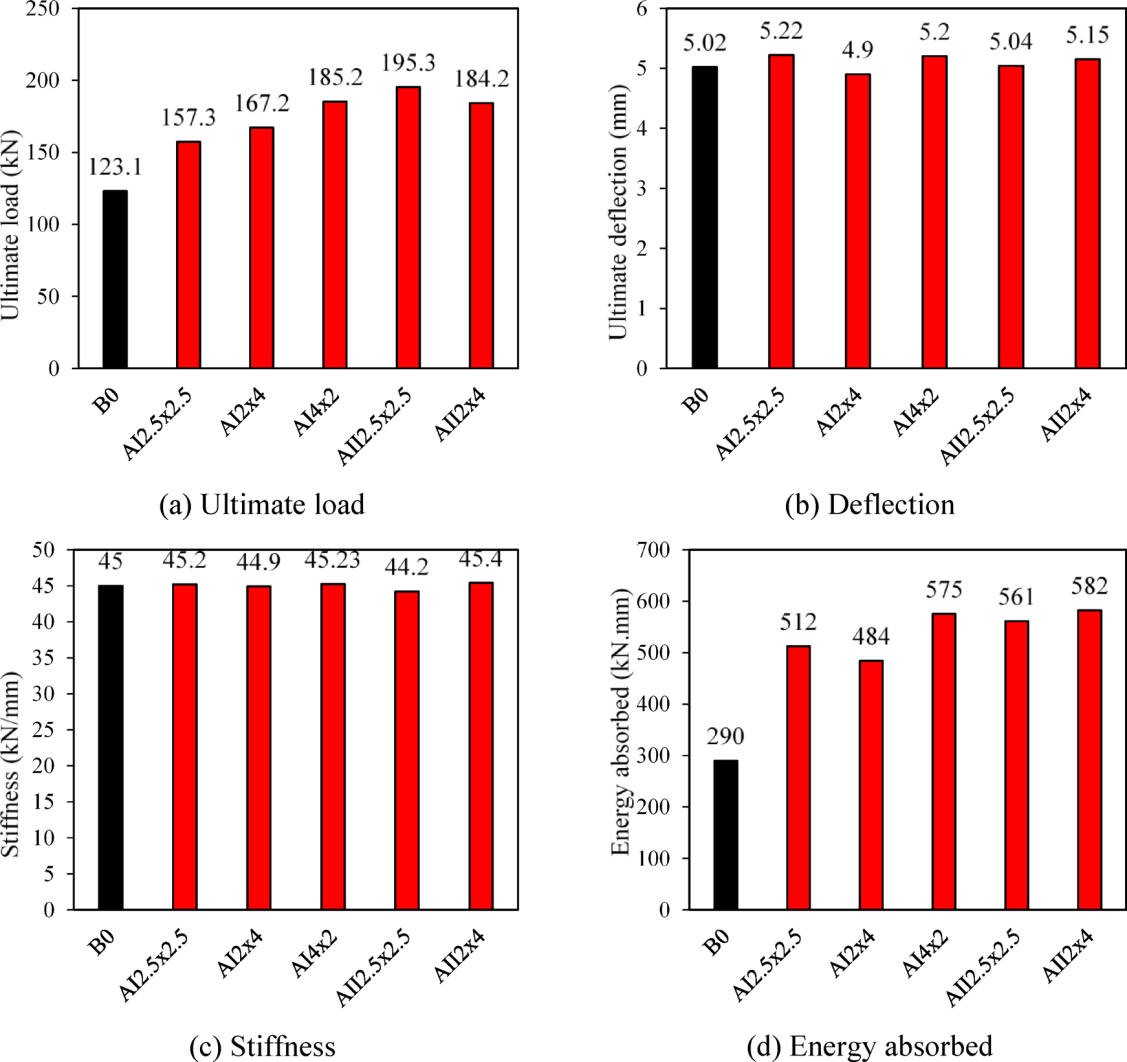
Effect of void reinforcement using ATs on performance of tested beams.

Group E examines the effect of increasing the aluminum tube area ratio (λa) on the structural response of voided RC beams (Fig. 16), in comparison with the solid control beam (B0). The inclusion of aluminum tubes within the voided regions led to consistent improvements in all performance parameters. At λa = 0.75% (AI2.5 × 2.5), the ${P}_{u}$ increased by 27.8% with minimal changes in stiffness and deflection, while EA rose significantly by 76.6%. The ductility index slightly improved to 1.30 (+ 3.17%), indicating that strength enhancement was achieved without compromising deformability. At λa = 0.9%, further strength gains were observed. Beam AI2 × 4 achieved a 35.8% increase in ${P}_{u}$ with nearly unchanged ductility (DI = 1.27), while AI4 × 2 exhibited the highest strength improvement in this subgroup (50.5%) and a corresponding increase in energy absorption (+ 98.3%). The DI values (≈1.30) confirm that changes in geometry had a limited but stable influence on ductility, while significantly improving strength and toughness. For higher λa values (1.5%–1.8%), beams AII2.5 × 2.5 and AII2 × 4 maintained this trend, with strength increasing up to 58.7% and EA approaching a 100% improvement. The ductility index remained nearly constant (1.27–1.30), indicating that the aluminum tubes mainly enhanced ${P}_{u}$ and EA rather than altering deformation capacity. Overall, increasing λa enhances confinement efficiency and internal stress redistribution in voided beams, resulting in significant improvements in strength and energy absorption, with stability in stiffness and ductility. The stable DI values indicate that aluminum tube reinforcement boosts structural efficiency while maintaining ductile performance.

#### Effect of number of voids reinforced with ATs (group B*)

**Fig. 17:**
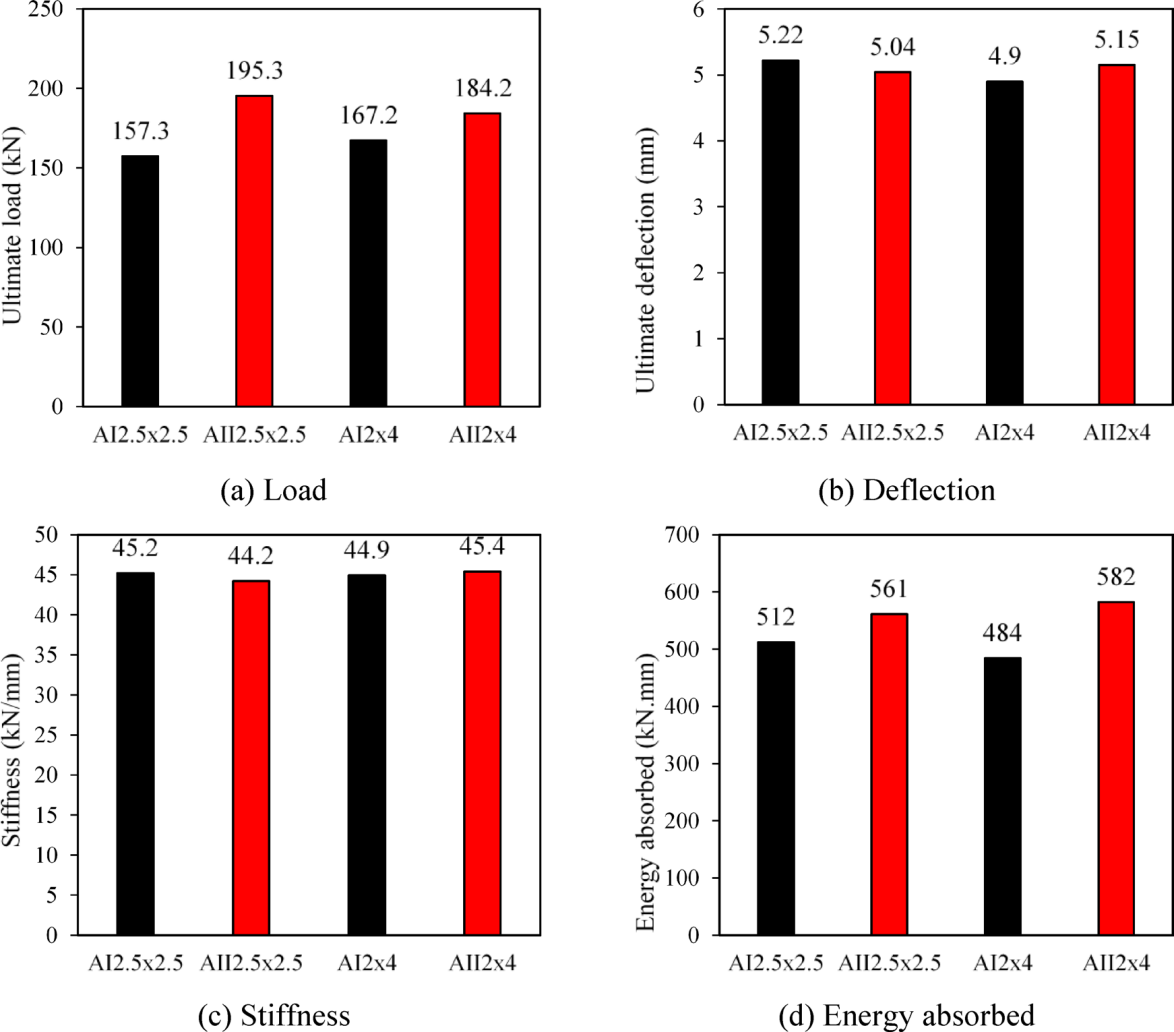
Effect of number of voids reinforced using ATs on performance of tested beams.

All beams in this group had identical aluminum tube (AT) geometry, while varying in the number of voids and total reinforcement ratio (λa), as shown in Fig. 17. Increasing the number of voids to two in AII2.5 × 2.5 (α = 6.2%, λa = 1.5%) enhanced structural capacity, with a 24.2% increase in ${P}_{u}$ and a 9.6% rise in EA. Despite the increased void ratio, stiffness and deflection remained nearly unchanged, while DI slightly decreased to 1.27 (− 2.3%), indicating a marginal reduction in ductility due to higher confinement demand, yet without compromising overall deformability. A similar response was observed for the 2 × 4 configuration. Beam AI2 × 4 (α = 4%, λa = 0.9%) showed improved strength (${P}_{u}$= 167.2 kN), while AII2 × 4 (α = 8%, λa = 1.8%) achieved an additional 10.2% increase in strength and 20.3% improvement in energy absorption. The DI values for both beams (1.27–1.30) confirm that ductility remained largely stable, with only minor variations linked to void expansion and reinforcement redistribution. Overall, increasing the number of AT-encased voids enhances load-carrying capacity and energy dissipation through cumulative confinement and improved stress redistribution around voids. Meanwhile, stiffness and deformation capacity remain relatively stable, and ductility shows only slight fluctuations (within ± 2.3%), as summarized in Table 5.

#### Effect of orientation of voids reinforced with ATs (group C*)

**Fig. 18:**
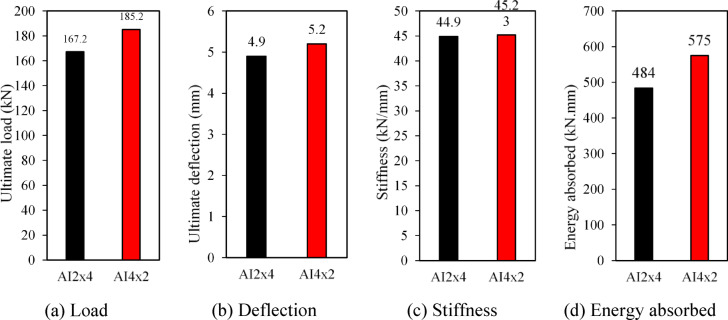
Effect of orientation of voids reinforced with ATs on performance of tested beams.

This group (C*) investigates the influence of void orientation on the behavior of AT-reinforced RC beams, while maintaining constant void-to-section ratio (α = 4%) and reinforcement ratio (λa = 0.9%) (Fig. 18). Changing the void orientation to horizontal in AI4 × 2 improved structural performance, with a 10.8% increase in ${P}_{u}$ and an 18.8% rise in EA. Stiffness remained nearly unchanged (+ 0.7%), while deflection slightly increased (+ 6.1%), indicating that elastic behavior was preserved while post-cracking performance improved. Consistently, the DI increased to 1.30 (+ 2.36%), indicating a modest enhancement in ductility compared with beam AI2 × 4. This improvement is attributed to the more efficient stress redistribution in the horizontal void configuration, which reduces stress concentration around void boundaries and enhances the interaction between concrete and embedded aluminum tubes. Consequently, crack propagation is delayed and energy dissipation capacity is improved without affecting initial stiffness.

## Conclusion

This study experimentally investigated the structural behavior of over—reinforced concrete (RC) beams with voids in the compression zone, reinforced with embedded aluminum tubes (ATs). A total of eleven RC beams—including one solid beam, five hollow beams without internal reinforcement, and five hollow beams reinforced with ATs—were tested under four-point bending to evaluate the effects of void ratio, number, orientation, and aluminum reinforcement on flexural performance. The key findings are summarized as follows:

1. Introducing voids in the compression zone led to a clear degradation in structural performance. Compared with the solid beam, hollow beams without ATs exhibited reductions of up to 36.5% in ultimate load capacity, 44% in stiffness, and 44.8% in energy absorption. Even a small void ratio (3.1%) caused a noticeable strength loss of about 18.7%, highlighting the sensitivity of over-reinforced sections to compression-zone discontinuities.
2. Increasing the number of voids at constant total void area intensified stress concentrations and accelerated deterioration, particularly for rectangular openings. Void orientation also influenced behavior; vertically oriented voids showed slightly better performance than horizontal ones due to improved alignment with compressive stress flow.
3. Embedding ATs within voids significantly enhanced structural performance, effectively compensating for strength loss and, in several cases, exceeding the capacity of hollow counterparts. Ultimate load increased by 57–135%, energy absorption by 126–264%, and ultimate deflection by up to 72% compared with unreinforced hollow beams.
4. Increasing the AT area ratio (0.75–1.8%) consistently improved post-cracking strength and energy dissipation without significantly affecting initial stiffness or ductility, indicating that strength enhancement was achieved without compromising deformation capacity.
5. For AT-reinforced beams, horizontal voids performed better than vertical ones, increasing ultimate load by 10.8% and energy absorption by 18.8% due to improved stress redistribution and composite action. Increasing the number of reinforced voids further enhanced performance, raising ultimate load by up to 24% and energy absorption by 20%.

For practical design, a compression-zone void ratio up to 8% combined with AT reinforcement is recommended, while an AT-to-section area ratio ≥ 0.75% is needed to maintain structural integrity. Serviceability checks (deflection and cracking) must be independently verified, as long-term behavior may differ from solid beams. Proper bonding and surface treatment of ATs are essential to ensure composite action. Reported strengths are laboratory-based; therefore, safety factors and code compliance must be applied, and prototype testing is advised for critical applications.

### Limitations and future recommendations

Although the present study provides comprehensive experimental insights into the flexural behavior of over-reinforced RC beams incorporating compression-zone voids and embedded aluminum tube reinforcement, several limitations should be recognized. These limitations are primarily associated with the instrumentation resolution, the depth of analytical interpretation, and the lack of advanced numerical validation. While the experimental program effectively captures the global structural response, ultimate capacity, and governing failure modes, it does not provide a detailed quantification of local response characteristics, including strain localization, crack initiation and propagation, and internal stress redistribution mechanisms. Furthermore, the interpretation of stress flow patterns associated with different void configurations and orientations was derived mainly from experimental evidence without complementary analytical or numerical corroboration. Although the findings suggest that horizontally oriented voids may exhibit improved alignment with principal compression stress trajectories, this observation requires further rigorous verification. In addition, the absence of parametric numerical simulations limits the ability to generalize the observed behavior and to systematically evaluate the influence of key geometric and material parameters. Accordingly, future research should integrate high-resolution experimental techniques, such as digital image correlation, with nonlinear finite element modeling and sectional analytical approaches to quantitatively assess stress transfer mechanisms, validate the experimental observations, and develop a more robust theoretical framework for the proposed structural system.

## Data Availability

The datasets used and/or analyzed during the current study available from the corresponding author on reason able request.
